# Mathematics Teachers’ Encouragement of Their Students’ Metacognitive Processes

**DOI:** 10.3390/ejihpe12090088

**Published:** 2022-09-01

**Authors:** Wajeeh Daher, Iman Hashash

**Affiliations:** Department of Educational Sciences, An-Najah National University, Nablus P.O. Box 7, Palestine

**Keywords:** mathematics teachers, students’ metacognition, questionnaire, gender, academic qualification, years of experience, planning, monitoring, regulating, evaluating

## Abstract

Researchers have conducted little research into teachers’ practices to encourage their students’ metacognition. The present research attempted to address this issue quantitatively by suggesting a questionnaire that measured teachers’ encouragement of students’ planning, monitoring, regulating, and evaluating. We present the results of the validity and reliability of the questionnaire. In addition, using a one-sample *t*-test, the results of the research revealed “normal”, “good”, and “very good” levels of teachers’ encouragement of their students’ metacognitive practices. The present research utilized an independent-sample *t*-test to investigate the significance of the difference in teachers’ metacognitive practices due to gender and to academic qualification. The results indicated that the metacognitive practices for male and female teachers were significantly different in planning and regulating, while the differences were not significant in monitoring and evaluating. In addition, the research results indicated that the participating teachers’ practices related to students’ metacognitive processes did not differ significantly due to the teachers’ academic qualification. When utilizing a one-way ANOVA test to investigate the significance of the difference in teachers’ metacognitive practices due to years of experience, this difference was not significant for any of the factors of metacognitive practices.

## 1. Introduction

Researchers have been interested in metacognitive processes since Flavell [1], but generally they have been interested in the processes of students (e.g., [2,3,4]). Studies have addressed the influence of metacognition on the cognitive aspect of students’ learning (e.g., [5]), the social aspect of students’ learning (e.g., [6]), the behavioral aspect of students’ learning [7], and the affective aspect of students’ learning (e.g., [6]). Lately, researchers addressed the preparation of prospective teachers in developing their metacognitive processes in problem solving (e.g., [8]) and in their design of mathematical activities (e.g., [9]). The present research addresses a different issue, that of teachers’ practices related to the metacognitive processes of their students in the mathematics classroom.

It was Flavell [1] who introduced the phrase “metacognition” to mean one’s awareness of, consideration of, and ability to control their own cognitive processes. Several definitions have been given for the term since then. Specifically, metacognition has come to be seen as self-reflection during the complex thinking process with which the learner is engaged during their learning activity, which includes planning the solution of the task, monitoring this solution, and evaluating progress [10]. It is the learner’s ability to be aware of, in control of, and monitor their learning processes. In addition, Davidson and Sternberg [11] defined metacognition as the processes of control, the function of which is planning, monitoring, and evaluating an individual’s performance in solving problems.

It is expected that any activity carried out by the learner to accomplish a specific learning task passes through two types of activities: cognitive activity to acquire or develop information and knowledge, and metacognitive activity that directs, organizes and evaluates the individual’s thinking. Therefore, knowledge and metacognition are two overlapping processes, and to distinguish between them, it was suggested by Shahbari et al. [12] that cognitive processes relate to activities such as reading, drawing, and computation, whereas metacognitive processes deal with planning, selecting appropriate strategies, and monitoring behaviors. Frith [13] pointed out that cognition is the processes by which knowledge is conceived, while metacognition means the individual’s knowledge of the processes they need in a situation, and that cognition means the strategies and processes that the individual uses to learn, while metacognition includes what the student knows about their awareness and ability to control knowledge.

In light of the foregoing, we can conclude that cognition and metacognition are only two complementary processes. Where knowledge is the entry point and the building block for metacognition, metacognition is the assistant to build and develop knowledge. The individual’s learning of metacognitive skills helps them to control their thinking, raising their level of awareness so that they can control it, direct it, and modify its course in the direction that brings them closer to achieving the goal. In addition, metacognitive thinking is one of the higher-order thinking skills, and is therefore used in the performance of mathematical tasks.

Below, we describe the four metacognitive skills that have been pointed out in the literature as the main metacognitive skills in learning and teaching in the classroom. 

### 1.1. Planning

The planning skill aims to form the stages of implementation of higher mental processes in order to control the thinking processes necessary to carry out the task and maintain the required outcome. This planning includes the activities that organize the learning processes, define the objectives of the learning process, state the problem in scientific formulation, and afterward select the appropriate strategy for solving the problem [14]. It also includes expecting obstacles and errors and ways to confront them, predicting results, and determining mechanisms to verify the work done.

### 1.2. Monitoring and Regulating

Monitoring and regulating involve an individual’s awareness of the actions they follow to organize the cognitive processes they plan to carry out and aims to detect errors or delays in the implementation process, as well as to verify the correct use of strategies and tools [15]. The skill of monitoring and regulating includes a number of activities: keeping the goal in focus, maintaining the sequence of steps in the thinking process, defining ways to achieve goals, knowing the appropriate time to move to the next act, choosing the appropriate processes for thinking, discovering errors and obstacles and overcoming them [16].

### 1.3. Evaluating

The evaluation skill aims to examine the extent to which the objectives of the cognitive process have been achieved and the individual’s evaluation of their learning processes, in addition to assessing the quality of planning the strategies used [17]. It includes judging the accuracy of results, the suitability of strategies with the steps used, and assessing the confrontation of errors and obstacles, in addition to evaluating the duration and effectiveness of the cognitive process plan and its implementation.

## 2. Literature Review

Researchers were interested in what affects metacognition, in addition to what is affected by metacognition. Suriyon et al. [18] found that open-approach-based teaching helped mathematics students exhibit metacognitive behaviors and abilities relevant to four teaching processes: (1) posing open-ended problems, (2) independent learning, (3) whole class discussion, and (4) conclusions of mathematical ideas. 

Schneider and Artelt [19], when reviewing the literature, found that declarative metacognition affected students’ performance in mathematics substantially. Moreover, metacognition-based teaching methods positively affected mathematics learners, including those with low mathematics performance.

Özçakir Sümen [20] found that metacognitive self-regulation predicted students’ mathematics achievement significantly, although metacognitive self-regulation did not mediate significantly between problem posing and students’ achievement. The previous findings agreed with Wang et al. [21], who found that metacognitive skills, interest, and self-control each uniquely predicted students’ engagement in mathematics learning. In addition, metacognitive skills interacted with interest and self-control to affect students’ engagement in mathematics learning. In addition, Akbar [22] found that the use of metacognitive skills positively affected students’ reasoning in the mathematics classroom. 

Few studies considered metacognition in teaching. Daher et al. [23] described an experiment in which they educated preservice teachers for metacognitive practices in problem solving as well as in designing mathematical activities. They concluded that to succeed in this preparation, the preservice teachers needed to experience the metacognitive skills both as learners and as teachers. In the two channels, they needed to negotiate how to use the metacognitive skills as they engaged in learning and in teaching. Moreover, Şeker and Engin [24] found that mathematics teachers considered metacognition an important compound phenomenon that positively affects students’ learning. Alzahrani [25] concluded that metacognition-based instruction of mathematics should be planned to improve the monitoring and regulation processes of students’ mathematical thinking in problem solving.

### Background Variables’ Influence on the Implementation of Metacognitive Skills

While many studies have been conducted on gender differences in metacognition and self-regulation skills, the results were unclear. The learning strategies of male students were more superficial than those of female students according to Niemivirta [26], and female students used more self-monitoring and setting goals than male students according to Bidjerano [27]. Liliana and Lavinia [28] found that both females and males used metacognitive processes in their learning.

Teachers’ academic qualifications seem to influence their practices. The results of a study by Manning et al. [29] demonstrated that higher teacher qualifications consistently correlated with a higher level of care and education for young children. In addition, some studies pointed at years of experience as influencing educational variables in the classroom. For example, Podolsky et al. [30] determined that during the careers of many teachers, those with more experience were more likely to succeed in measures other than test scores.

## 3. Research Rationale and Goals

Research that addressed the level and function of metacognition in the classroom, and specifically in the mathematics classroom, has mainly addressed students’ learning processes [31]. The present research differed in that it addressed teachers’ practices regarding the encouragement of students’ metacognitive processes in the mathematics classroom. Specifically, it suggested a teachers’ questionnaire for evaluating their practices in four metacognitive processes: planning, monitoring, regulating, and evaluating. These processes were agreed upon as the main metacognitive processes [32]. In suggesting this questionnaire, the present research provided quantitative methods for addressing the metacognitive practices of encouraging students’ metacognitive processes not only in the mathematics classroom, but in other disciplines as well. 

When suggesting the questionnaire, we also intended to use it in order to investigate the level of middle school teachers’ practices related to the encouragement of their students’ metacognitive processes. In addition, we used the questionnaire to verify whether the teachers’ practices regarding the encouragement of their students’ metacognitive processes differed due to their gender, academic qualification, and years of experience. 

## 4. Methodology

This research followed the descriptive research design, which provides information about the relevant variables [33]. It was conducted in the governmental middle schools in Ramallah Governate in the second trimester of the academic year 2020–2021.

### 4.1. Research Sample

The research sample consisted of 260 middle school mathematics teachers. Table 1 describes the frequency of the values for each of the background variables: gender, academic qualification, and years of experience. 

As we found differences in the level of the components of teachers’ metacognitive skills, we excluded diploma and Ph.D. participants, as they were low in comparison with the other academic qualifications. 

### 4.2. Data-Collecting Tools

Depending on the literature [34,35,36], we developed a questionnaire that measured teachers’ use of metacognitive skills in the mathematics classroom. The questionnaire specifically measured four metacognitive skills: planning (5 items), monitoring (7 items), regulating (5 items), and evaluating (5 items). Each item was scored using the following Likert scale response options: 1 = rarely, 2 = sometimes, 3 = frequently, and 4 = always.

Table 2 provides the descriptive statistics for each of the questionnaire items; the items are shown according to their status as metacognitive components.

### 4.3. The Statistical Tests’ Assumptions

The test for the normality of the data was not performed because the number of respondents exceeded 30 or 40 [37], so a violation of the normality assumption would not lead to major problems in computing the scores for the parametric tests [38]. 

The tests for the homogeneity of variance resulted in insignificant Levene’s tests over each of the background variables; i.e., gender, academic qualification, and experience, which proved that the scores of teachers’ metacognitive practices were homogenous over these variables. 

### 4.4. Data Analysis

The factorability of the questionnaire was examined using an exploratory factor analysis followed by a confirmatory factor analysis. 

As the scores for the teachers’ metacognitive practices were homogenous over the entire group, a one-sample *t*-test was used to find the significance of the level of teachers’ metacognitive practices. 

**Figure 1:**
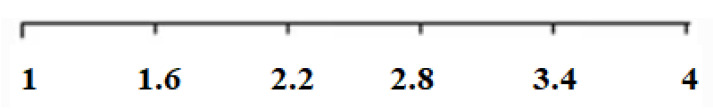
Intervals related to the metacognitive practice scores.

To evaluate the level of teachers’ metacognitive practices, we compared them with the “good metacognitive practice score” and the “normal metacognitive practice score.” We computed the good metacognitive practice score by dividing 3 (3 units between 1, the lowest score of any item, and 4, the highest score of any item) by 5 (5 intervals), resulting in 0.60; thus, we obtained the points related to the metacognitive practice intervals presented in Figure 1. We considered the two middle points (2.2 and 2.8) to be the “normal metacognitive practice score” and the “good metacognitive practice score”, respectively. In addition, the score 3.4 was considered the “very good metacognitive practice score.”

The scores for teachers’ metacognitive practices were homogenous over the research groups divided according to the background variables, except planning over years of experience. The present research carried out an independent-sample *t*-test to determine the significance of the differences in teachers’ metacognitive practices due to the variables of gender and academic qualification. Moreover, the present research carried out an ANOVA test to investigate the significance of the differences in teachers’ metacognitive practices due to years of experience. The Brown–Forsythe test was used for the planning variable.

## 5. Results

Initially, a study was conducted on the factorability of 30 items related to teachers’ use of metacognitive skills in which several factors for determining the factorability of the items were examined. Firstly, all items were conditioned to have a correlation of no less than 0.4 with no less than one other item, meaning good factorability. This left us with 22 items: three factors with 5 items each and one factor with 7 items. Then, a Kaiser–Meyer–Olkin sample adequacy measure of 0.865 was calculated for the 22 items, which was higher than the proposed value of 0.6. In addition, the Bartlett’s test of sphericity showed significance (χ^2^(253) = 2340.321, *p* < 0.001), which indicated that the factor model was appropriate [39]. 

Moreover, the diagonals of the anti-image correlation matrix were all above 0.7, and lastly, the communalities of each item were all higher than 0.4, which asserted that every item shared some characteristics with the rest of the items. Given the previous results, a principal factor analysis seemed suitable for all 22 items. Table 3 shows the factor loadings for the items in the above scale (N = 260); these loadings were based on an Oblimin rotation as part of a principal axis analysis. 

**Figure 2:**
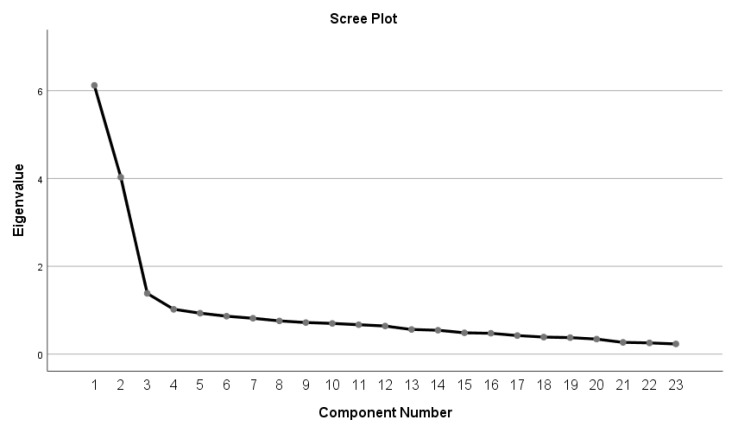
The scree plot: factorability of the 22 items into four factors.

We identified and calculated the composite scores for the factors in the scale. The eigenvalues showed that the factors explained 26.611%, 17.518%, 6.018%, and 4.437% of the variance of evaluating, monitoring, planning, and regulating, respectively. According to this factorability, the four factors explained 54.58% of the variance in the scores of the teachers’ metacognitive skills. A scree plot (see Figure 2 below) supported the suggested factorability of the 22 items into four factors.

Correlations between the four factors are presented in Table 4.

The correlations in Table 4 were low and moderate relationships, indicating their fit as factors of the construct of metacognition. 

### 5.1. Validity and Reliability Analysis

Validity and reliability were investigated for the extracted factors. For the purpose of validating the questionnaire, the first version was presented to experts in mathematics for analysis and therefore verification of its validity to collect data. After the experts’ verification, the recommended corrections were made to the scale, which resulted in the present 22-item, 4-point Likert-type rating scale. To investigate reliability, the present research computed Cronbach’s alpha for each of the four factors of teachers’ use of metacognitive skills. This computation resulted in 0.797 for evaluation, 0.864 for monitoring, 0.730 for planning, and 0.763 for regulating. These reliability values implied a good reliability of the extracted factors [40]. 

Statistics were performed to compute the model fit related to a confirmatory factor analysis (CFA) of the questionnaire. The χ^2^ value for the questionnaire was 477.175 (d.f. = 203, *p* = 0.134), so the relative χ^2^ was (CMIN/d.f. = 2.351). Moreover, the RMSEA index was 0.04 (90% CI = 0.023; 0.062), which succeeded in supporting the fit of the questionnaire. Bentler’s CFI was 0.987, which further indicated that the proposed model fit the questionnaire. The NFI, TLI, GFI, and AGFI were 0.987, 0.988, 0.990, and 0.968, respectively, also indicating that the proposed questionnaire had a good fit.

A parallel analysis was carried out to validate the number of factors of the metacognitive construct using the rawpar.sps script suggested by O’Connor [41]. The raw data were permuted to produce 1000 datasets using a factor analysis [42], which suggested a four-factor solution. 

### 5.2. The Level of Teachers’ Metacognitive Practices

A one-sample *t*-test was used to find the significance of the level of teachers’ metacognitive practices as compared with 2.8 (the good metacognitive practice score) and 3.4 (the very good metacognitive practice score). Table 5 shows the results.

Table 5 shows that the level of planning was significantly ‘very good’. It also shows that although the mean score for monitoring was more than 2.8 (the good metacognitive score), it was not significantly so, and thus it was significantly ‘normal’. Furthermore, although the mean scores for evaluating and regulating were more than 3.2 (the very good metacognitive score), they were not significantly so, but they were significantly ‘good’. 

### 5.3. The Difference between Teachers’ Metacognitive Practices due to Gender

As the scores of the factors of teachers’ metacognitive practices were not normal over the gender variable, the present research carried out the Mann–Whitney U test to investigate the significance of the differences in teachers’ metacognitive practices due to gender. The computations demonstrated that the metacognitive practices for male and female teachers were significantly different for planning and monitoring, while the differences were not significant for regulating and evaluating. Table 6 shows the results.

Table 6 shows that male teachers had lower scores in the four metacognitive practices, but these differences were significant only in planning and regulating. 

### 5.4. The Difference between Teachers’ Metacognitive Practices due to Academic Qualification

The present research carried out an independent-sample *t*-test to determine the significance of the differences in teachers’ metacognitive practices due to the academic qualification variable. Table 7 shows the means, standard deviations, and results of the independent-sample *t*-test for the factors of teachers’ metacognitive practices related to academic qualifications in the mathematics classroom.

Table 7 shows that the mean scores of teachers’ metacognitive practices due to academic qualification were not significantly different for any of the factors of metacognitive practices.

### 5.5. The Difference between Teachers’ Metacognitive Practices Due to Years of Experience

As the scores of the factors of teachers’ metacognitive practices were homogenous over the years of experience variable, the present research carried out an ANOVA test to determine the significance of the differences in teachers’ metacognitive practices due to years of experience. Table 8 shows the means, standard deviations, and results of the ANOVA test for the factors of teachers’ metacognitive practices, related to years of experience, in the mathematics classroom.

Table 8 shows that though there were differences in teachers’ metacognitive practices due to years of experience, these differences were not significant for any of the factors of metacognitive practices.

## 6. Discussion and Recommendations

Studies on teachers’ use of teaching-metacognitive processes are not extensive [43]. Particularly, the empirical issue of teachers’ metacognitive practices related to encouraging their students’ metacognitive processes is still little studied. One approach to develop such empirical research is through utilizing quantitative means such as questionnaires, which help in evaluating different aspects of teachers’ metacognitive processes, such as the level of these processes. The present paper suggested a questionnaire that evaluated four metacognitive practices of teachers: planning, monitoring, regulating, and evaluating. The four suggested factors explained 54.58% of the variance in the entire teachers’ metacognitive score. A four-factor scale solved the issue of scales with just one or two factors, which might not have offered a precise image of the construct [44], while a scale with four factors did. In addition, the four metacognitive factors have been accepted by different researchers as the main metacognitive practices ([1,8]).

The challenges of the modern era require the nourishing of a self-reliant student who is capable of self-learning. Teachers involved in the study seemed to be aware of these challenges, but not equally. The research results indicated “normal”, “good”, and “very good” levels of teachers’ encouragement of their students’ metacognitive practices. Thus, the results implied that the participating mathematics teachers were aware, to some extent, of the importance of these metacognitive practices, not only to the modern era, but specifically to problem solving [1]. 

Despite teachers’ awareness of planning, regulating, and evaluating, less attention was paid to monitoring, which is an important metacognitive process that enriches the cognitive processes of problem solving [45]. This result concerning the level of monitoring indicated that teachers need to pay more attention to monitoring; training workshops for teachers could contribute to an increase in this attention. Researchers have argued that monitoring is a pre-condition for regulating that involves selecting cognitive strategies, setting task goals, and allocating study time, which allows an individual to effectively manage their cognitive processes [46]. The previous argument stressed the importance of making teachers aware of the importance of encouraging students to use monitoring in their problem solving. 

No clear findings were reported in the previous literature regarding the differences in metacognitive practices between male and female students [26,27,28]. Here, the research results implied that female teachers had significantly greater scores in planning and regulating. This was in line with Peña-López [47], who argued that compared to male teachers, female teachers believe teaching to be less about the transmission of knowledge and more about structure and student-centered practices, and are more cooperative with colleagues. This belief that teaching is about structure and student-centered practices would encourage an emphasis on metacognitive practices. 

The research results indicated that the participating teachers’ practices related to students’ metacognitive processes did not differ significantly due to the teachers’ academic qualifications. This insignificant result could be due to the similar curriculum with which the teachers were engaged. In addition, this agreed with previous studies that reported that academic qualifications did not result in significant differences in educational variables related to teachers as their beliefs (e.g., [45]). At the same time, the results did not agree with studies that pointed at academic qualifications as influencing teachers’ practices. One such study was that of Manning et al. [29] who, depending on the review of research, concluded that a higher level of teacher qualification consistently correlated with a higher quality of early childhood care and education, as demonstrated by the results.

The research results indicated that the participating teachers’ practices related to students’ metacognitive processes did not differ significantly due to the teachers’ years of experience. These results did not agree with studies that indicated differences in educational variables in the classroom due to teachers’ years of experience. For example, Podolsky et al. [30] determined that during the careers of many teachers, those with more experience were more likely to succeed in measures other than test scores. In addition, they do not agree with studies that reported that specific experiences influenced educational variables. For example, Daher [48] reported that teachers pointed at experience in the choice of tools as influencing the use of tools in promoting students’ creativity. 

## 7. Conclusions and Limitations

The present research intended to suggest a questionnaire for teachers’ encouragement of their students’ metacognitive practices. The questionnaire included four metacognitive practices: planning, monitoring, regulating, and evaluating. This questionnaire adds to the international literature, as the current literature regarding teachers’ encouragement of their students’ metacognitive practices is still scarce. The questionnaire needs to be validated in various contexts, as here it was validated in a Palestinian mathematics classroom. This was one limitation of the study, but this limitation will be overcome when the questionnaire is validated in other disciplines and cultural contexts.

The present research examined different issues related to teachers’ encouragement of their students’ practices. Here too, the cultural context could be a limitation, and thus these issues should be studied in other cultural contexts, school levels, and school classroom disciplines.

The present study took one step further regarding the issue of teachers’ encouragement of their students’ metacognitive practices. It calls for further attempts to study this issue. 

## Figures and Tables

**Table 1:** Frequencies of the values of each of the background variables.

Variable	Variable Values	N
Gender	Male	80
	Female	180
Academic qualification	B.A.	166
	M.A.	73
Experience	5 years or less	85
	More than 5 years up to 10 years	57
	More than 10 years up to 15 years	32
	More than 15 years	86

**Table 2:** Descriptive statistics of the questionnaire items (N = 260).

		M	SD	Skewness
Item 1	I ask the students to summarize what they did while solving a task	3.26	0.811	−0.819
Item 2	I encourage the students to assess their understanding of the task.	3.28	0.725	−0.844
Item 3	I encourage the students to assess how far they have accomplished the objectives of the task.	3.35	0.707	−0.957
Item 4	When the students finish the solution, I discuss with them what they learned during the solution of the task.	3.37	0.683	−0.632
Item 5	When the students finish the solution, I discuss with them how to overcome the difficulties they encountered.	3.40	0.665	−0.753
Item 6	I encourage the students to discuss the effectiveness of the procedures used in solving the task.	3.05	1.006	−0.811
Item 7	I encourage the students to find methods of dealing with errors.	2.99	1.008	−0.661
Item 8	I encourage the students to discuss their solutions with their colleagues.	2.92	1.047	−0.561
Item 9	I ask the students to describe the steps they took while performing the educational task.	2.95	1.004	−0.606
Item 10	I encourage the students to set a specific time to complete the task.	2.90	.979	−0.487
Item 11	I encourage the students to stop and examine their solution process.	2.58	.908	−0.090
Item 12	I direct the students to change solution strategies even when the current strategy does not solve the task.	2.69	1.001	−0.190
Item 13	I encourage the students to organize the givens of a problem before solving it.	3.68	0.566	−1.697
Item 14	I encourage the students to partition the problem into smaller parts.	3.52	0.660	−1.192
Item 15	I encourage the students to read the problem before solving it.	3.64	00.590	−1.407
Item 16	I encourage the students to determine the problem elements before solving it.	3.45	0.671	−0.992
Item 17	I encourage the students to keep the purpose of the educational task in their focus.	3.60	0.570	−1.108
Item 18	I encourage the students to devote time for identifying ways that assist them in overcoming difficulties in problem solving.	3.33	0.764	−0.841
Item 19	I encourage the students to be aware of all possible options for solving the task.	3.24	0.684	−0.416
Item 20	I encourage the students to discuss the elements of the material that help solve related problems.	3.23	0.746	−0.740
Item 21	I discuss with the students to how to make proceed in accurate problem solving.	3.40	0.726	−1.032
Item 22	I encourage the students to review the learning material that could direct their solution.	3.40	0.687	−0.785

**Table 3:** Factor loading of the items.

Item		Component			
		EV	MO	PL	RE
Item 1	I ask the students to summarize what they did while solving a task.	0.842			
Item 2	I encourage the students to assess their understanding of the task.	0.750			
Item 3	I encourage the students to assess how far they have accomplished the objectives of the task.	0.676			
Item 4	At the end of the solution, I discuss with the students what they learned during the solution of the task.	0.528			
Item 5	At the end of the solution, I discuss with the students how to overcome the difficulties they encountered.	0.444			
Item 6	I encourage the students to discuss the effectiveness of the procedures used in solving the task.		0.849		
Item 7	I encourage the students to find methods of dealing with errors.		0.821		
Item 8	I encourage the students to discuss their solutions with their colleagues.		0.780		
Item 9	I ask the students to describe the steps they took while performing the educational task.		0.777		
Item 10	I encourage the students to set a specific time to complete the task.		0.752		
Item 11	I encourage the students to stop and examine their solution process.		0.608		
Item 12	I direct the students to change solution strategies even when the current strategy does not solve the task.		0.533		
Item 13	I encourage the students to organize the givens of a problem before solving it.			0.750	
Item 14	I encourage the students to partition the problem into smaller parts.			0.662	
Item 15	I encourage the students to read the problem before solving it.			0.624	
Item 16	I encourage the students to determine the problem elements before solving it.			0.579	
Item 17	I encourage the students to keep the purpose of the educational task in their focus.			0.487	
Item 18	I encourage the students to devote time for identifying ways that assist them in overcoming difficulties in problem solving.				−0.667
Item 19	I encourage the students to be aware of all possible options for solving the task.				−0.660
Item 20	I encourage the students to discuss the elements of the material that help solve related problems.				−0.654
Item 21	I discuss with the students how to proceed keeping accurate problem solving.				−0.522
Item 22	I encourage the students to review the learning material that could direct their solution.				−0.510

MO = monitoring; EV = evaluating; RE = regulating; PL = planning.

**Table 4:** Correlations between the factors of metacognition.

	Monitoring	Planning	Regulation
Evaluation	0.315	0.517 **	0.562 **
Monitoring		0.404	0.327
Planning			0.568 **

** *p* < 0.01.

**Table 5:** Means, standard deviations, and one-sample *t*-test for the level of metacognitive practices scores.

	N	Mean	SD	t_2.8_	*p*	t_3.4_	*p*
Planning	260	3.5277	0.43656	26.878	0.000	4.716	0.000
Monitoring	260	2.8665	0.73831	1.452	0.148	−11.652	0.000
Evaluating	260	3.3392	0.54070	16.081	0.000	−1.812	0.071
Regulating	260	3.3623	0.50477	17.963	0.000	−1.204	0.230

**Table 6:** Mean of ranks for the factors of teachers’ metacognitive practices in the mathematics classroom (N = 180 for female and N = 80 for male).

	Gender	N	Mean	Std. Deviation	t	*p*
Planning	Female	180	3.5878	0.40564	30.396	0.001
	Male	80	3.3925	0.47462		
Monitoring	Female	180	2.9246	0.74796	10.914	0.057
	Male	80	2.7357	0.70321		
Evaluating	Female	180	3.3511	0.53758	0.531	0.596
	Male	80	3.3125	0.55012		
Regulating	Female	180	3.4267	0.47527	30.136	0.002
	Male	80	3.2175	0.54116		

**Table 7:** Independent-sample *t*-test for academic qualification (N = 166 for B.A teachers and N = 73 for M.A. teachers).

	Qualification	Mean	SD	t	*p*
Planning	Bachelor	3.539	0.422	00.073	0.942
	Master	3.534	0.416		
Monitoring	Bachelor	2.871	0.705	−00.570	0.569
	Master	2.930	0.791		
Evaluating	Bachelor	3.351	0.516	−00.001	0.999
	Master	3.351	0.533		
Regulating	Bachelor	3.352	0.511	−00.653	0.514
	Master	3.397	0.459		

**Table 8:** ANOVA for teachers’ metacognitive practices over years of experience in the mathematics classroom (N = 85 for 5 years or less, N = 57 for greater than 5 years up to 10 years, N = 32 for greater than 10 years up to 15 years, and N = 86 for greater than 15 years).

		Mean	SD	95% Confidence Interval for Mean		F	*p*
				Lower Bound	Upper Bound		
Planning	5 years or less	30.461	0.388	30.378	30.545	0.272	287
	More than 5 to 10 years	30.509	0.414	30.399	30.619		
	More than 10 to 15 years	30.563	0.546	30.366	30.759		
	More than 15 years	30.593	0.448	30.497	30.689		
	Total	30.528	0.4366	30.474	30.581		
Monitoring	5 years or less	20.808	0.746	20.648	20.969	10.435	0.233
	More than 5 to 10 years	20.820	0.785	20.611	30.028		
	More than 10 to 15 years	30.112	0.724	20.851	30.373		
	More than 15 years	20.864	0.698	20.714	30.014		
	Total	20.867	0.738	20.776	20.957		
Evaluating	5 years or less	30.332	0.478	30.229	30.435	00.445	0.721
	More than 5 to 10 years	30.361	0.547	30.216	30.507		
	More than 10 to 15 years	30.244	0.650	30.009	30.478		
	More than 15 years	30.367	0.556	30.248	30.487		
	Total	30.339	0.5410	30.273	30.405		
Regulating	5 years or less	30.287	0.493	30.181	30.393	10.757	0.156
	More than 5 to 10 years	30.330	0.49207	30.199	30.460		
	More than 10 to 15 years	30.363	0.58018	30.153	30.572		
	More than 15 years	30.458	0.48784	30.354	30.563		
	Total	30.362	0.50477	30.301	30.424		
